# Research Progress in Computational Fluid Dynamics Simulations of Membrane Distillation Processes: A Review

**DOI:** 10.3390/membranes11070513

**Published:** 2021-07-07

**Authors:** Long Chen, Binxin Wu

**Affiliations:** College of Biosystems Engineering and Food Science, Zhejiang University, Hangzhou 310058, China; chenlong1988@zju.edu.cn

**Keywords:** membrane distillation, computational fluid dynamics, heat transfer, mass transfer

## Abstract

Membrane distillation (MD) can be used in drinking water treatment, such as seawater desalination, ultra-pure water production, chemical substances concentration, removal or recovery of volatile solutes in an aqueous solution, concentration of fruit juice or liquid food, and wastewater treatment. However, there is still much work to do to determine appropriate industrial implementation. MD processes refer to thermally driven transport of vapor through non-wetted porous hydrophobic membranes, which use the vapor pressure difference between the two sides of the membrane pores as the driving force. Recently, computational fluid dynamics (CFD) simulation has been widely used in MD process analysis, such as MD mechanism and characteristics analysis, membrane module development, preparing novel membranes, etc. A series of related research results have been achieved, including the solutions of temperature/concentration polarization and permeate flux enhancement. In this article, the research of CFD applications in MD progress is reviewed, including the applications of CFD in the mechanism and characteristics analysis of different MD structures, in the design and optimization of membrane modules, and in the preparation and characteristics analysis of novel membranes. The physical phenomena and geometric structures have been greatly simplified in most CFD simulations of MD processes, so there still is much work to do in this field in the future. A great deal of attention has been paid to the hydrodynamics and heat transfer in the channels of MD modules, as well as the optimization of these modules. However, the study of momentum transfer, heat, and mass transfer mechanisms in membrane pores is rarely involved. These projects should be combined with mass transfer, heat transfer and momentum transfer for more comprehensive and in-depth research. In most CFD simulations of MD processes, some physical phenomena, such as surface diffusion, which occur on the membrane surface and have an important guiding significance for the preparation of novel membranes to be further studied, are also ignored. As a result, although CFD simulation has been widely used in MD process modeling already, there are still some problems remaining, which should be studied in the future. It can be predicted that more complex mechanisms, such as permeable wall conditions, fouling dynamics, and multiple ionic component diffusion, will be included in the CFD modeling of MD processes. Furthermore, users’ developed routines for MD processes will also be incorporated into the existing commercial or open source CFD software packages.

## 1. Introduction

Membrane distillation (MD) is a membrane separation technology, in which a hydrophobic micro porous membrane is used as a medium, and the steam pressure difference between the two sides is used as the mass transfer driving force. It is a process in which momentum, heat and mass are coupled to each other and transferred simultaneously. The principle of MD is that under certain conditions, the solution to be separated at a certain temperature is transported to the hot side of the surface of the hydrophobic micro porous membrane, while the cold side of the membrane is directly or indirectly in contact with the cold media. After evaporation, the volatile components in the feed solution on the hot side will be transferred to the cold side through the membrane pores, driven by the partial pressure difference of steam, and will be collected after condensation on the cold side. However, the non-volatile components are blocked in the hot side due to the hydrophobicity of the membrane, to enable the separation or purification of the solution to be separated [1].

**Figure 1:**
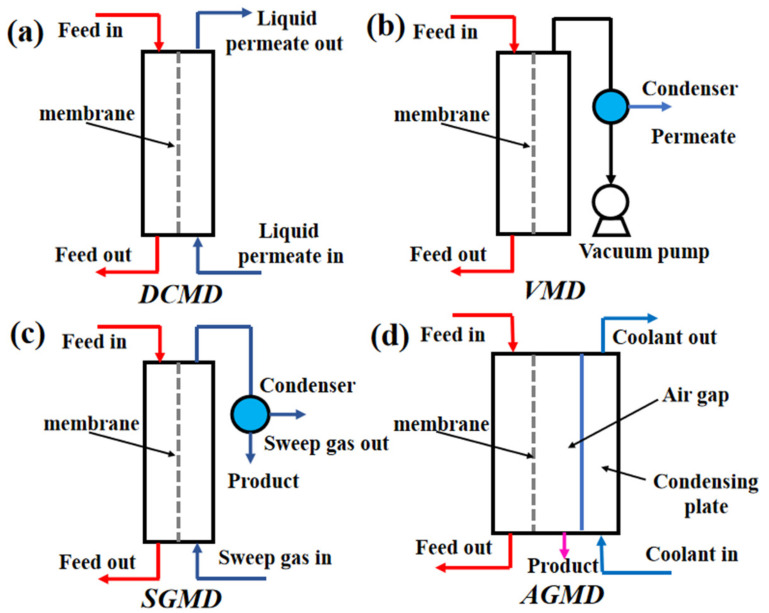
Four types of MD Process [3]. Reproduced with permission from Elsevier, 2011. (**a**) Direct contact membrane distillation (DCMD); (**b**) Vacuum membrane distillation (VMD); (**c**) Sweep gas membrane distillation (SGMD); (**d**) Air gap membrane distillation (AGMD).

Since the introduction of MD in 1963 [2], four basic mechanism types have been labeled according to the different ways in which the volatile components are condensed on the permeate side of the membrane: direct contact membrane distillation (DCMD), air gap membrane distillation (AGMD), sweep gas membrane distillation (SGMD), and vacuum membrane distillation (VMD). The four basic types are shown in Figure 1. There are also some new configurations, including permeate gap membrane distillation, liquid gap membrane distillation, material gap membrane distillation, water-cooled membrane distillation and air-cooled membrane distillation, with these configurations being derived from the four basic ones. Therefore, this review deals mainly with the basic MD types.

**Figure 2:**
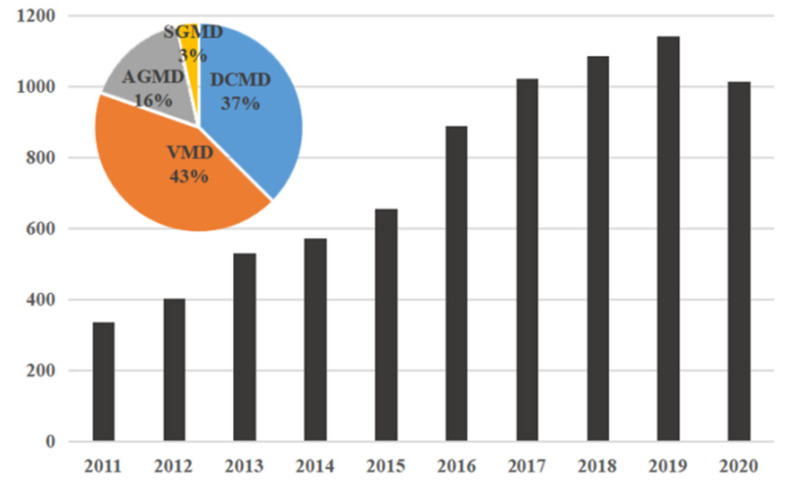
Growth in the number of refereed papers on membrane distillation from 2011 to 2020.

Initially, DCMD was the most extensively studied, however more recently, the research results related to VMD are increasing, and now exceed DCMD [3]. In recent years, with the increasing conflicts between population, resources and environment, the number of research results on MD has also increased significantly (Figure 2). In 2017, a paper was published in *Nature Nanotechnology* on the use of the porous carbon nanotube polymer composites as self-heating membranes to improve the transmembrane driving force [4]. In this paper, a high one-way recovery rate of nearly 100% was achieved by heating high salinity brine directly on the membrane distillation element, far exceeding the standard MD recovery limit, bringing MD technology back to the field of view. To be compared with other separation technology, MD has many advantages: it can be operated at a low operating temperature, low operation pressure; the volatile substances withholding rate is 100% in theory, it is not affected by material solutions pressure for separation [5], etc. Moreover, the MD technology can be used in seawater water desalination, recovery of volatile solute removal and recycling in aqueous solution, the concentration of liquid food, wastewater treatment and other fields, showing that it has a very broad application prospect. 

MD is a process mainly suited to applications in which water is the major component present in the feed solution [3], such as the desalination of seawater, the preparation of ultra-pure water, and the treatment of industrial wastewater. Although MD is well suited to the environmental, chemical, petrochemical, food, pharmaceutical and biotechnology industries, it is still in the experimental stage and has not yet achieved full industrial application. The main reasons are:

(1) the problem of continuous decrease of permeate flux;
(2) lacking practical experiences in the application of large-scale MD equipment required by the project.

Membrane fouling and temperature polarization are the main reasons for the decrease of permeate flux. Membrane fouling refers to the phenomenon that contaminants block the membrane holes, or the membrane holes are wetted during MD process. Temperature polarization is a phenomenon which is caused by the existence of a hot boundary layer, that is, the temperature of the hot side membrane surface is lower than that of the hot side main body, while the temperature of the cold side membrane surface is higher than that of the cold side main body. The existence of this phenomenon reduces the effective driving force of temperature difference on both sides of the membrane, which leads to the decrease of the driving force of mass transfer across the membrane and the low heat transfer rate. In other words, the temperature difference between the bodies on both sides of the membrane is not all used for the vaporization of the feed solution, thus leading to the decrease of the flux efficiency of the membrane. 

MD has not been applied on a large-scale due to the lack of an effective method for calculating energy loss and the high cost of preparing hydrophobic porous membranes. At present, the research of MD processes based on CFD simulations mainly focus on the analysis of novel membranes and membrane characteristics, the design optimizations analysis of membrane components, and the analysis of different structural characteristics. However, research on momentum transfer and heat and mass transfer mechanisms in membrane holes rarely occurs. 

In this article, the latest progress and existing problems in MD technology based on CFD are introduced, and the prospect of CFD in promoting the industrialization and applications of MD technology are put forward.

## 2. Applications of CFD in Analysis of Mechanisms and Characteristics of Different MD Structures

Mathematical models can be systematically used in the analysis and optimization of a MD process [6]. It can be widely used in the estimation of the temperature polarization coefficient [7], outlet temperature prediction [8], permeability flux prediction [9,10] and another research needs in characterizing the MD process. CFD is based on the three conservation laws of mass, momentum and energy, by solving the three equations of fluid mechanics to get the expression of variables [11]. This section mainly describes the applications of CFD technology in the analysis of the mechanisms and characteristics of different MD structures.

### 2.1. Applications of CFD in Analysis of Direct Contact Membrane Distillation (DCMD)

In DCMD, as shown in Figure 1, the feed solution to be separated is directly in contact with the hot side surface of the membrane, while the permeation side is directly in contact with the cold side surface of the membrane [12]. Driven by the steam pressure difference, the feed solution evaporates on the hot side of the membrane and reaches the cold side surface of the membrane through the pores, then condenses on the permeation side, thus, the separation process is completed [3]. Due to the advantages of simple structure, stable permeate flux, high gain output ratio (GOP) and easy to separate volatiles, DCMD has the largest number of related studies. Compared with other MD types, high temperature polarization is easily generated in DCMD, and the feed concentration and pore wetting have greater influences on the permeate flux [13,14].

**Figure 3:**
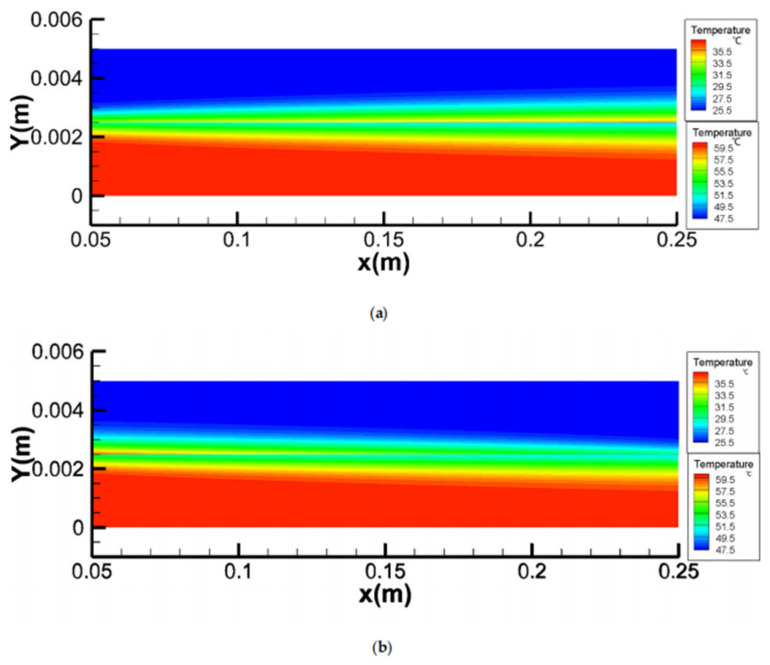
Temperature fields for co- and counter-current operations [15]. Reproduced with permission from MDPI, 2020. Different color scales were used in the permeate and feed channels, T_feed_ = 60 °C, T_permeate_ = 25 °C, v_feed_ = v_permeate_ = 0.15 m/s, concentration of feed = 24.2 wt.%) (**a**) co-current operation; (**b**) counter-current operation.

**Figure 4:**
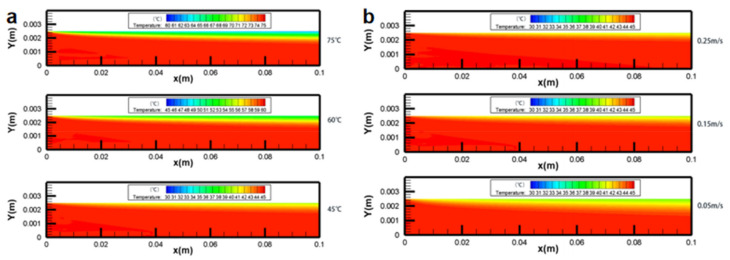
Contours of temperature distribution [15]. Reproduced with permission from MDPI, 2020. (**a**) in different T_feed_ (v_feed_, v_permeate_ = 0.15 m/s, T_permeate_ = 25 °C, cf = 24.2 wt.%); (**b**) in different vfeed, vpermeate (T_feed_ = 45 °C, T_permeate_ = 25 °C, c_feed_ = 24.2 wt.%).

The effects of feed temperature (T_feed_), feed velocity (v_feed_), and permeate velocity (v_permeate_) on temperature polarization were presented [15]. The authors established a simulation model of heat and mass transfer coupling on the membrane surface through User-defined function (UDF), and studied in detail the effects of temperature, inlet velocity (Figure 3 and Figure 4) and solution concentration on the evaporation efficiency of the membrane module under co-current and counter-current operations. The results show that the temperature polarization increases with an increase of the feed temperature and a decrease of the feed velocity and permeate velocity; the counter-current operation is better than the co-current operation in most cases, except for relatively low NaCl concentrations or for a sufficiently long module length.

A nonlinear dynamic model to capture the outlet temperature of DCMD was proposed [8,14], and the effectiveness of the model was verified with experimental data. The results show that the DCMD process has a strong convective heat transfer mechanism. The main reason is that the temperature polarization and the Biot number (Bi) in DCMD processes are high. The membrane conduction resistance is dominant at very low flow rates, which limits the heat transfer at the membrane interface, thus affecting the process performance.

### 2.2. Applications of CFD in Analysis of Vacuum Membrane Distillation (VMD)

In the VMD, as shown in Figure 1, a vacuum pump is added on the permeation side and the vacuum applied is lower than the saturation pressure of volatile component separation [3]. Unlike DCMD, the condensation process of VMD occurs outside the membrane module. Although the pore size of VMD is smaller than that of other structures, it also increases the risk of membrane wetting. Due to the advantages of VMD, such as the high permeate flux, the ability to effectively separate volatile and aromatic compounds, low temperature polarization, and that the pores are not wetted at the distillate side [13], research related to VMD has continued to increase in recent years, and now exceeds DCMD research.

**Figure 5:**
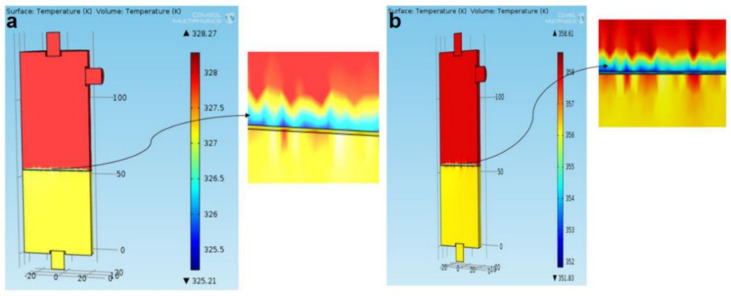
Temperature contour inside membrane module [16]. Reproduced with permission from Elsevier, 2020. (Flow rate = 5l pm, Vacuum degree = 750 mmHg, Initial dye concentration = 30 ppm); (**a**) at 55 °C feed temperature; (**b**) at 85 °C feed temperature.

A 3D CFD model of the VMD process using COMSOL multi-physical field simulation software was established [16], which could predict the permeate flux and surface temperature in the process. The simulations show that the heat and mass transfer coupling phenomena exist at the interface between the feed solution and the membrane surface. In this CFD model, the authors describe the transfer equations of momentum, heat, and mass. As shown in Figure 5, the authors first determined the temperature and concentration distribution inside the membrane and membrane module by using CFD simulation, and calculated the influence of theoretical permeate flux and different operating conditions on flow rate, temperature and vacuum degree by using the convective heat transfer mechanism of the porous membrane. Then the CFD model was used to analyze the effect of concentration on permeate flux. Finally, the interface temperature was determined and the temperature polarization coefficient (TPC) for different operating parameters was estimated. It has been noted that the feed temperature and vacuum degree were found to significantly affect operating variables for a higher permeate flux and minimum specific energy consumption. The considerable reduction in the TPC value from 0.81 to 0.48 was discovered while raising feed temperature from 25 °C to 85 °C, indicating a significant reduction in heat transfer resistance, resulting in a remarkable increase in permeate flux. 

**Figure 6:**
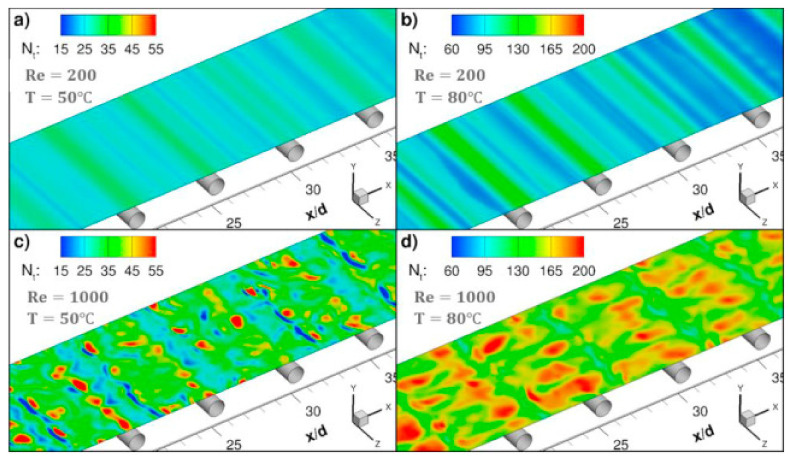
Instantaneous contours of the local mass flux, N_t_ (kg/m^2^h), through the top membrane surface at a non-dimensional time τ_i_ with the addition of filaments [17]. Reproduced with permission from Elsevier, 2019. (**a**) Reynolds Number (Re) = 200, T = 50 °C; (**b**) Re = 200, T = 80 °C; (**c**) Re=1000, T = 50 °C; (**d**) Re = 1000, T = 80 °C.

In the literature [17], a sub-grid scale large eddy simulation was adapted to carry out 3D transient CFD simulation with wall adaptive local eddy viscosity. The local concentration, temperature and permeate flux were coupled on the membrane surface (Figure 6), and the diffusion rate of water vapor through the membrane was predicted by Knudsen and the viscous diffusion mechanism. The predicted value is in good agreement with the measured value, which verifies the correctness of the model. The small-scale eddies induced by the presence of spacer filaments promote mixing in the module, thus the temperature and concentration polarization are alleviated and the water vapor flux is immensely improved. The insertions of filaments in the feed channel increase the water permeate rate by more than 50% at higher feed flow rates and inlet temperatures. The pressure drop by the spacer reduces the allowable module length by one order of magnitude, but the module length increases two folds at feed temperature 80 °C. Even though the power consumption of the module containing the filaments is increased, the addition of filaments is strongly recommended since the required power for the process could be supplied from readily available low-grade heat sources.

### 2.3. Applications of CFD in Analysis of Air Gap Membrane Distillation (AGMD)

In AGMD, an air gap is added between the membrane surface and the condensation surface. The evaporated gas needs to pass through the pores and the air gap sequentially, and finally condenses on the cold side of the membrane module [3]. In this structure, the probability of membrane fouling is minimal and the heat loss is less than the other MD types. However, due to disadvantages including the complex structure, membrane module being too difficult to design, mass transfer resistance having been increased by adding an air gap, GOP being the lowest versus the other MD types, multiple model variables, and modeling being too difficult [15], relevant studies on AGMD are much fewer than those on DCMD and VMD.

**Figure 7:**
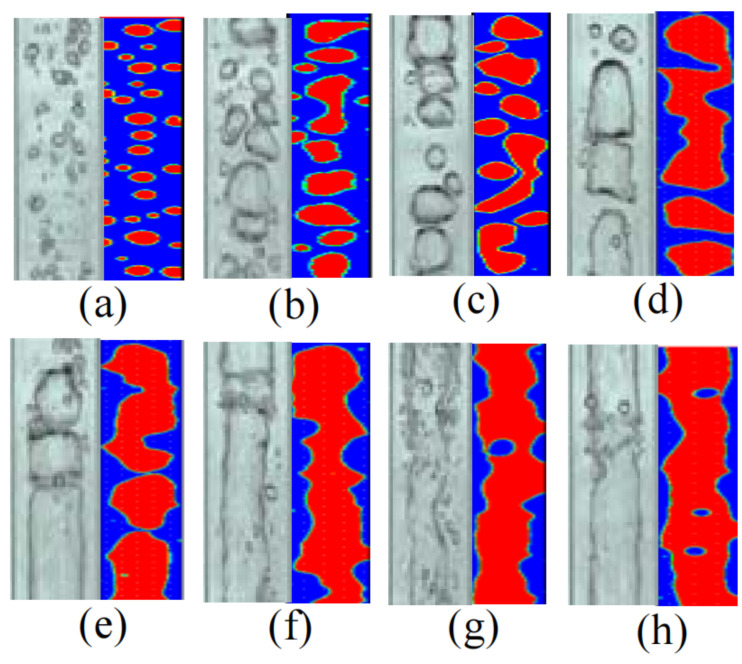
Effects of Nitrogen flow on two-phase flow pattern and model validation [18]. Reproduced with permission from Higher Education Press, 2019. (**a**) 10 lph, (**b**) 20 lph, (**c**) 30 lph (**d**) 40 lph, (**e**) 50 lph, (**f**) 60 lph, (**g**) 70 lph, (**h**) 80 lph.

**Figure 8:**
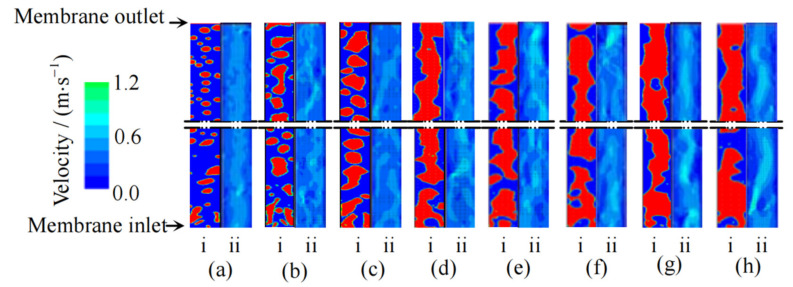
Effects of gas holdup on flow pattern and velocity [18]. Reproduced with permission from Higher Education Press, 2019. Gas holdup: (**a**) 0.20; (**b**) 0.33; (**c**) 0.43; (**d**) 0.50; (**e**) 0.56; (**f**) 0.60; (**g**) 0.64; (**h**) 0.67. (i). Transient flow pattern (red/light-gas phase; blue/dark-liquid phase); (ii). Velocity.

Pan et al. [18,19,20,21,22] carried out a series of studies on AGMD characteristics strengthened by gas–liquid two-phase flow (Figure 7 and Figure 8), and obtained the rules of membrane wall shear stress, patterns of the gas–liquid two-phase flow, velocity distributions and turbulence intensities by using CFD simulation method. The simulation results can be used to qualitatively analyze the disturbance of gas relative to liquid phase and the interaction between two phases in the process of two-phase flow enhanced MD, which provides a reference for further study of the mechanism of two-phase flow enhanced mass transfer. Studies have shown that, in the AGMD process, the permeate flux can be increased by 4.70 and 5.46 times as much as that of DCMD after nitrogen and low-pressure water vapor are introduced. However, under the same experimental conditions, the permeation fluxes of water vapor with the same flow rate are all greater than that of nitrogen. The gas–liquid two-phase flow pattern with the same flow rate of nitrogen and low-pressure water vapor is obviously different from that of the feed liquid. In the momentum transfer process of AGMD, the turbulence intensity obviously increases. In the process of heat transfer of AGMD, the effect of the total temperature difference driving force is improved and the concentration polarization phenomenon is obviously weakened (the maximum temperature polarization factor of nitrogen and low-pressure water vapor can reach 1.64 times and 2.37 times that of DCMD; The concentration polarization coefficient can be reduced to 0.62 times and 0.22 times as low as that without filling any bubble gas).

### 2.4. Applications of CFD in Analysis of Sweep Gas Membrane Distillation (SGMD) 

In SGMD, as shown in Figure 1, an inert gas sweeps through the permeate side and carries the vapor outside the membrane module for condensation [3]. This structure has the lowest temperature polarization and no risk of membrane pores wetting on the distillate side, so it is a good prospect to concentrate aqueous solutions. However, due to disadvantages such as, the structure brings relatively complex, difficult heat recovery, low permeate flux, and the need to provide dry and clean sweep air [13], the relevant studies on SGMD are the fewest of all the MD types. Modeling of SGMD is usually achieved by fitting the heat and mass transfer coefficients and using empirical correlations.

**Figure 9:**
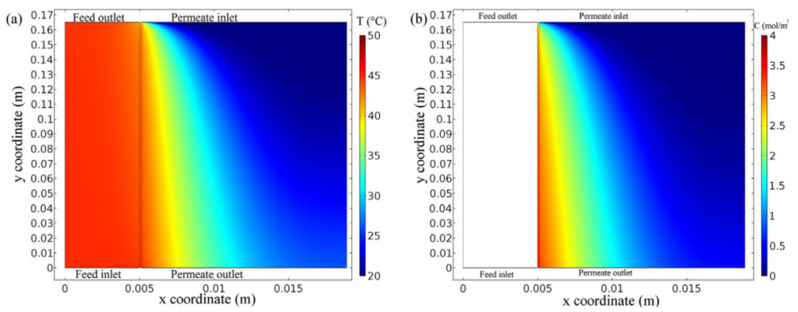
Results of the simulation of the flat sheet SGMD module [23]. Reproduced with permission from Elsevier, 2018. (**a**) The temperature distribution in all domains and (**b**) distribution of the water vapor concentration in the membrane media and permeate channel (v_Feed_ = 5.6 × 10^−3^ m/s, v_Permeate_ = 9.3 × 10^−2^ m/s, T_Feed_ = 45 °C, T_Permeate_ = 20 °C).

A CFD-based general prediction model for SGMD was introduced in the literature [23]. As shown in Figure 9, this model allows the simulation of a hollow-fiber membrane module and a flat sheet membrane module under a wide range of operate conditions with less input data and no need for empirical parameters or experimental data. In this prediction model, the momentum, mass and heat balance in SGMD processes are described by partial differential equations, algebraic equations and ordinary differential equations.

The influences of membrane properties of SGMD on permeate flux, temperature and concentration polarization characteristics of membrane modules in a seawater desalination process investigated by CFD were presented [24]. When the pore size is determined, the effects of porosity and membrane thickness on permeate flux are different. The results show that the membrane thickness has a great influence on the SGMD performance, while the membrane porosity has a little influence on the SGMD performance.

## 3. Applications of CFD in Optimization Analysis of Membrane Module Designs

The type of membrane module is the most important unit in a MD system. The advantages and disadvantages of a membrane module structure will directly affect the separation efficiency and economic performance of MD [25]. This section mainly reviews the influences of the design and optimization of membrane modules on the MD process, and the applications of CFD in design. Commonly used industrial membrane modules include flat sheet membrane, tubular membrane, hollow-fiber membrane, and spiral wound membrane modules. CFD technology plays a key role in the design and optimization of membrane modules [26,27]. Experiments show that optimization of membrane modules and flow channels can significantly increase permeate flux, reduce membrane fouling and temperature polarization [28,29].

**Figure 10:**
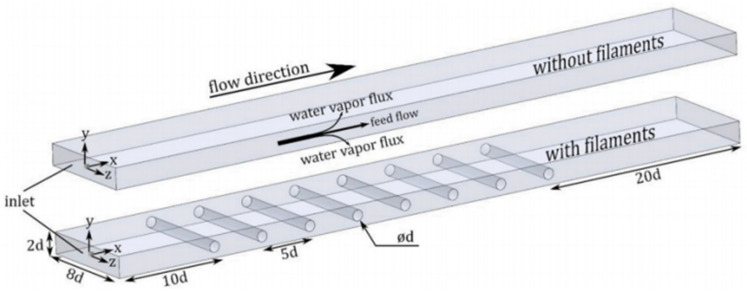
Schematic of Flow Geometry without and with filaments [17]. Reproduced with permission from Elsevier, 2019.

**Figure 11:**
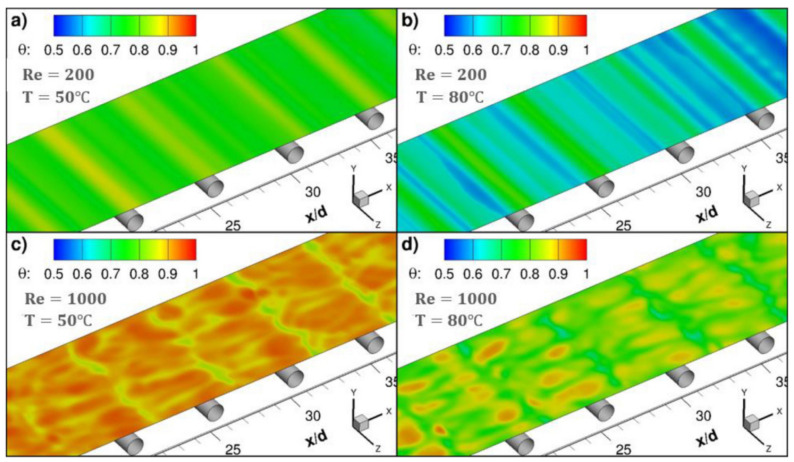
Instantaneous contours of the temperature polarization coefficient, θ, on the top membrane surface at a non-dimensional time τ_i_ with the addition of filaments [17]. Reproduced with permission from Elsevier, 2019. (**a**) Re = 200, T = 50 °C; (**b**) Re = 200, T = 80 °C; (**c**) Re = 1000, T = 50 °C; (**d**) Re = 1000, T = 80 °C.

**Figure 12:**
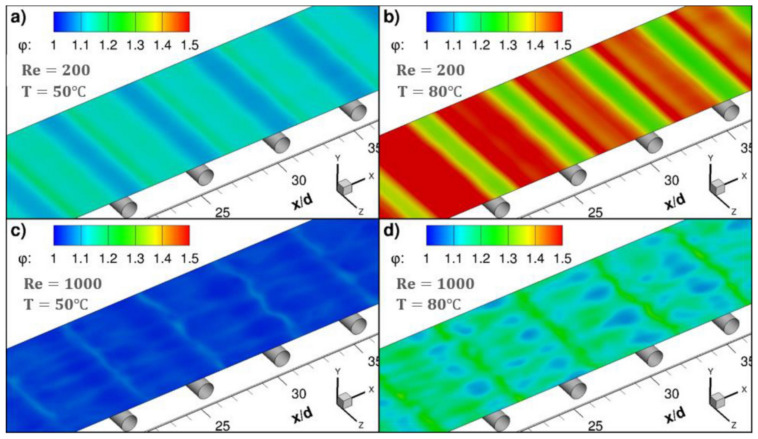
Instantaneous contours of the concentration polarization coefficient, φ, on the top membrane surface at a non-dimensional time τ_i_ with the addition of filaments [17]. Reproduced with permission from Elsevier, 2019. (**a**) Re = 200, T = 50 °C; (**b**) Re = 200, T = 80 °C; (**c**) Re = 1000, T = 50 °C; (**d**) Re = 1000, T = 80 °C.

Similar to other membrane processes, CFD techniques are widely used for optimization analysis of membrane module designs, such as reverse osmosis and electrodialysis. Through CFD simulations and experiments (Figure 10), the small-scale eddy currents caused by the presence of spacers can promote liquid mixing in MD membrane modules, thereby reducing temperature and concentration polarization (Figure 11 and Figure 12) and increasing permeate flux [17]. At higher feed flow and inlet temperatures, the water penetration rate can be increased by more than 50% by inserting spacers into the feed passage. The results show that the pressure drop from the spacers can reduce the length of the membrane module by an order of magnitude, but the length of the membrane module increases by two times when the feed temperature is equal to 80 °C. Although the addition of spacer filaments in the membrane module will lead to increased power consumption, but considering that the power required by the process can be provided from a waste heat source, this method is a feasible way to improve the permeate flux.

**Figure 13:**
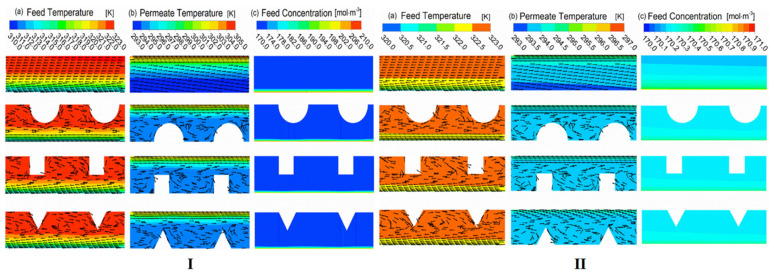
Local flow field visualization for various baffled modules [30]. Reproduced with permission from Elsevier, 2019. (**I**) under the laminar flow at 0.05 m/s (Re_Feed_ = 358; Re_Permeate_ = 200); (**II**) under the turbulent flow at 0.55 m/s (Re_Feed_ = 3935; Re_Permeate_ = 2196).

The performance of DCMD can be improved by adding a baffle to the inlet shell [30]. The optimization channel is designed as a regular baffle with different shapes at different characteristic lengths. The authors calculated and compared; the heat transfer coefficient, mass transfer coefficient, temperature polarization coefficient, concentration polarization coefficient, mass flux, thermal efficiency and power consumption of the original module and the optimized one. The results show that the structure modification can inhibit the temperature polarization and the concentration polarization phenomenon, so as to increase the water production. However, it should be noted that the presence of baffles also leads to additional power consumption. Under laminar flow conditions (Figure 13I), the water production performance can be significantly improved by adding baffles in the shell. However, under turbulent conditions (Figure 13II), the water production performance is not as good as laminar flow conditions, due to the huge increase of hydrodynamic loss.

**Figure 14:**
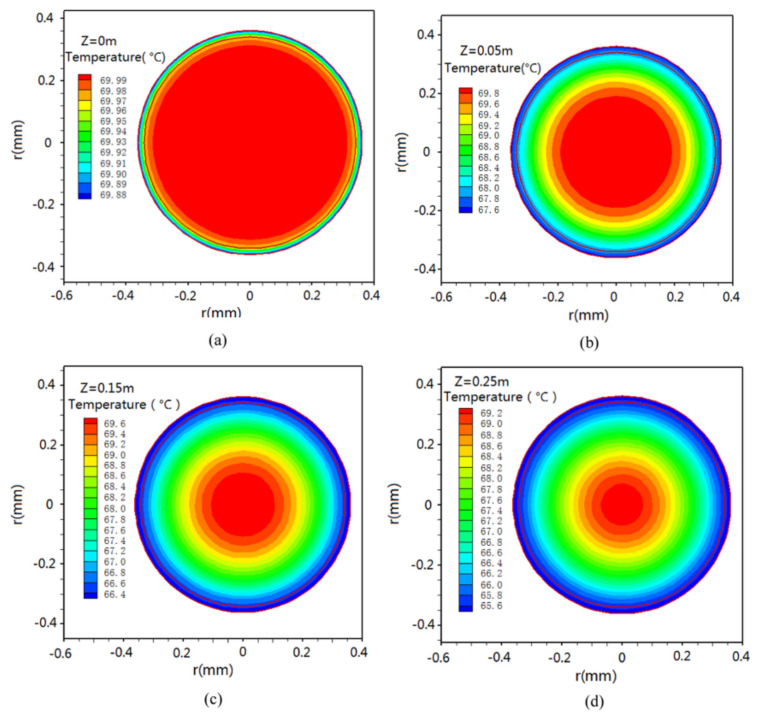
Temperature distributions on different cross sections of a fiber [31]. Reproduced with permission from Elsevier, 2016. z = 0 m (**a**), 0.05 m (**b**), 0.15 m (**c**), 0.25 m (**d**) (T_fi_ = 70 °C, υ_fi_ = 1.60 m/s, Pv = 3 kPa, L = 0.25 m).

The heat and mass transfer process of VMD hollow-fiber membrane modules in laminar flow using CFD were simulated [31], as shown in Figure 14. By combining latent heat with the energy conservation equation and experimental data, a 3D VMD model was established. On this basis, the effects of operating conditions and module sizes on; local temperature, heat transfer coefficient, temperature polarization coefficient, heat mass flux and total thermal efficiency of feed flow in fiber tubes and fiber shell under vacuum conditions were analyzed. The results show that the thermal efficiency varies with the feed temperatures and feed velocities, and thus the temperature polarization phenomenon is more obvious at high feed temperatures and low feed velocities.

A 3D model of DCMD for investigating five different spacer layouts in membrane modules was built [32]. The experimental results show that the spacer layout has a significant effect on the efficiency of the membrane module, and the existence of the spacers in the membrane module increases the pressure drop and increases the heat transfer coefficient by about two times.

**Figure 15:**
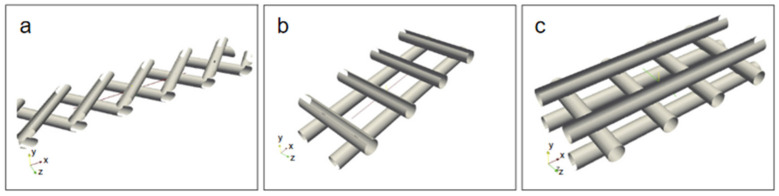
Spacer geometries [33]. Reproduced with permission from Elsevier, 2013. (**a**) spacer type 1; (**b**) spacer type 2; (**c**) spacer type 3.

**Figure 16:**
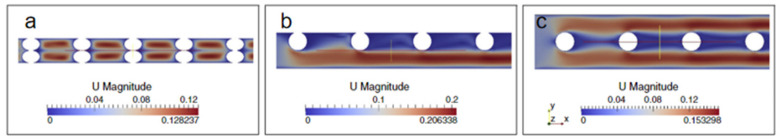
Velocity magnitude (in m/s) color maps on a z-normal plane through center of the domain [33]. Reproduced with permission from Elsevier, 2013. (**a**) spacer type 1; (**b**) spacer type 2; (**c**) spacer type 3.

**Figure 17:**
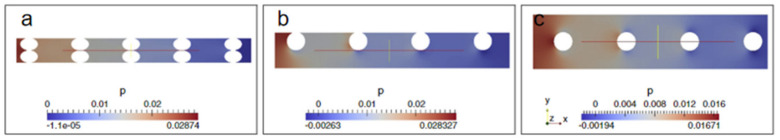
Pressure (in kPa) color maps on a z-normal plane through center of the domain [33]. Reproduced with permission from Elsevier, 2013. (**a**) spacer type 1; (**b**) spacer type 2; (**c**) spacer type 3.

Understanding hydrodynamic and heat transfer conditions and their effects on temperature polarization and pressure drop is also crucial for the optimal designs of MD modules. In literature [33], an open source CFD code base OpenFOAM was adapted to simulate the 3D momentum and heat transfer in the feed channels of DCMD, and discuss the influences of three different types of spacers (Figure 15, Figure 16 and Figure 17) on the hydrodynamics and heat transfer in the channels. The effect of the geometric characteristics of the partition on temperature polarization and pressure drop is highlighted.

The heat and mass transfer mechanism of hollow-fiber membrane modules with and without annular baffles in DCMD processes was investigated by CFD [34]. The experimental results show that a higher operating temperature can improve the heat and mass transfer and MD thermal efficiency even under relatively low temperature differences across the membrane.

The effects of baffle orientation, inlet velocity and fiber spacing on the shear stress distribution and temperature polarization of the membrane module were presented [35]. The CFD simulation results show that the orientation of the baffles influences the temperature polarization and heat transfer rate. If the filaments contact the upper or lower surface of the membrane, the temperature polarization is very high, which results in a lower heat transfer rate. When these filaments are separated from the membrane, the temperature polarization decreases. In the separation mode, the shear stress is higher, the TPC and the local value of the shear stress are more uniform, which makes this special orientation more suitable for the membrane module.

An intermittent operation mode with 3D printed spiral baffle and super hydrophobic membrane coating was proved to improve the MD performance [36]. The experimental results show that improving the heat and mass transfer and enhancing the hydrophobicity of the membrane will help the MD system to become a feasible choice for industrial wastewater treatment.

Spacers are an important component of a membrane module, which is the channel of feed and permeation flow. Good feed spacer positioning helps to improve permeability. The effects of inlet velocity, filament orientation and spacing on heat transfer were investigated [37]. The temperature polarization was used as a parameter to evaluate the heat transfer performance, and the shear stress and temperature polarization coefficient under different interval orientations in the DCMD process were calculated. The results show that the studied parameters have important effects on temperature polarization and shear stress. The permeate flux and thermal efficiency can be improved by increasing the flow rate and inlet temperature, and decreasing the length of the membrane module. Moreover, the temperature polarization coefficient distribution of the staggered filaments is more uniform, which indicates that this orientation is more suitable for enhancing the heat transfer in the MD process.

Under laminar flow, the water production can be significantly improved by attaching baffles in the channel shells, which is not useful under the turbulent flow due to the huge amount of hydrodynamic loss [38]. The mass transfer coefficient is similar for different baffled modules. The impacts of baffles on the water production lies in the transmembrane partial vapor pressure difference. Baffles impact the concentration polarization coefficient (CPC) and the temperature polarization coefficient (TPC), thus the transmembrane partial vapor pressure difference.

There are five new hollow-fiber module structures for DCMD processes designed and manufactured [39], including structured straight fiber, crimped fiber, central feed tube, spacer winding, and spacer braided fiber. The performance of the module was evaluated with a permeability flux experiment, fluid dynamics study, flow distribution tracer response test and heat transfer analysis. Compared with the conventional module, the permeate flux of the novel modules are increased by 53–92%, among which the interval braided module has the best performance. Except for structured straight fiber modules, all the new fluxes are independent of the feed flow rate. The membrane modules with undulating membrane surfaces (crimped fibers and spacer braided fibers) increased fluxes in the laminar flow region by more than 300%. Finally, the results of NaCl tracer response measurements demonstrate that the improved filaments geometry or arrangement can provide effective boundary layer surface renewal and a more uniform flow distribution. Among them, the curled fiber component has the advantage of minimum temperature polarization effect, which increases the penetration flux.

A heat recovery of the flat sheet membrane-hollowfiber exchanger (FSM-HFE) was simulated by CFD [40,41]. The fluid temperature distribution in the model was investigated under different feed temperatures, flow rates and operating vacuums, and the parameter distributions were obtained, which laid a foundation for further optimizations of the module internal structure.

Li et al. [42,43,44] carried out a series of studies on module optimizations of VMD, simulated and compared feed solution distribution devices with different structures by using CFD technology, and optimized the structural designs of feed solution distributors according to the calculation results. Finally, three optimal distribution devices are obtained; the plate type distributor, the arc type distributor and the pyramid type distributor. The experimental results show that the applications of the distributor significantly increase the permeate flux of the membrane module.

## 4. Applications of CFD in Preparation of Novel Membranes and Analysis of Membrane Characteristics

The membrane is the core part of the membrane module. This section concentrates on the applications and developments of CFD technology in hydrophobic porous membrane in MD processes. The current applications of CFD technology in research on membrane characteristics are summarized. The hydrophobic porous membranes used in the MD process include; Polytetrafluoroethylene (PTFE), Polypropylene (PP), Polyethylene (PE), Polyvinylidenfluoride (PVDF), etc. At present, flat and hollow-fiber membranes are the usual membrane structures [45]. PP and PTFE are widely used in MD, and it is difficult to distinguish them with the naked eye. 

The main factors affecting membrane performance include, membrane porosity, average pore size, membrane thickness, etc. Among them, membrane porosity has a great influence on permeate flux, water production ratio and thermal efficiency. Increasing the porosity and decreasing the membrane thickness and thermal conductivity can help to improve the water production ratio [46]. The larger the average pores size of the membrane, the higher the nucleation frequency and growth rate of membrane fouling, and the less conducive to the continuous operation of the MD process [47]. MD membranes should have good thermal stability and chemical stability, so they are not easily corroded by the feed solution. It should have good mechanical strength, so as to extend the service life of the membrane. It should have a lower thermal conductivity, so as to reduce the energy loss caused by thermal conductivity in the process of MD. Therefore, the selection of a MD membrane needs to strike a balance between low thermal conductivity and high permeate flux. To reduce thermal conductivity loss, thicker membranes should be selected. While to improve permeability flux, thinner membranes should be selected with a large pores size, low pore tortuosity, high porosity and thinner thickness [25].

As with other membrane separation processes, membrane fouling is still an unsolved problem for MD processes. Due to the differences in membrane structures, design and operating conditions, the membrane fouling mechanism of MD may be different than that from the pressure-driven mechanism. In order to solve the membrane fouling problem correctly, it is necessary to understand the fouling formation and mechanism of MD [48]. To solve the membrane fouling problem, investigators generally start from the interaction force between pollutants and hydrophobic membranes to modify the surface of hydrophobic membranes, and prepare the total hydrophobic membranes and Janus composite membranes, so as to avoid the adsorption of pollutants on the membrane surface and reduce the risk of wetting the pores. Appropriate membrane post-treatment measures can also restore the membrane performance [49]. In the MD process, the greater the TPC is, the smaller the actual steam pressure difference between the two sides relative to that of the main feed liquid. The greater the CPC is, the greater the difference between the membrane surface concentration and the main feed liquid concentration is, and the more serious the concentration polarization phenomenon is. Concentration polarization will directly affect the steam pressure on both sides, which will also make it easier for membrane fouling to appear on the membrane surface. It should be noted that the modification effect can only reduce the adhesion between the membrane and pollutants, making it easy for the pollutant to fall off or difficult to stay on the membrane surface, so the optimization of operating conditions is very critical [47]. CFD technology can be used to evaluate membrane fouling and distributions, study the optimization of membrane parameters, optimize operating conditions, and determine the mechanism of multi-phase heat and mass transfer across the membrane, which is conducive to the development of novel and more efficient membranes. It is worth mentioning that self-heating membranes are currently a hot research topic in the preparation of novel MD membranes [4,50].

The optimal membrane thickness of PVDF and PTFE was studied [15]. The results show that the optimal membrane thickness is in the range of 10–20 µm, which relates to the selected materials, pore size distribution and the maximum permeability flux under the operating conditions. The membrane materials with low thermal conductivity and high porosity obtain a higher permeation flux and a larger optimal membrane thickness, while the optimal membrane thickness can be reduced by increasing the feeding speed or temperature.

The influences of the characteristics of porous membranes on the desalination in VMD were experimentally studied [51]. They carried out the experiment in a rectangular chlorinated polyvinyl chloride cell. The authors used Knudsen diffusion model and a dusty-gas model to predict the mass transfer coefficients of different PTFE and PVDF membranes, and combined with experiments, studied the changes of the mass transfer coefficients of PVDF and PTFE flat sheet membrane modules under different vacuum levels and different feed velocities when the temperature of hot seawater varied between 65 °C and 85 °C. Their predicted value is very close to the measured value of the experimental data.

Haidari et al. [52] proposed a new approach for studying membrane fouling. The authors used CFD to model and simulate the spacers filling the channels of the membrane, compared the pressure drop, time and space velocity diagrams of channels filled with spacers and empty channels of the same size, to determine the influences of the feed spacing on the hydraulic conditions, and determined the probability of membrane fouling through the velocity distribution obtained by the particle image velocimetry experiment.

**Figure 18:**
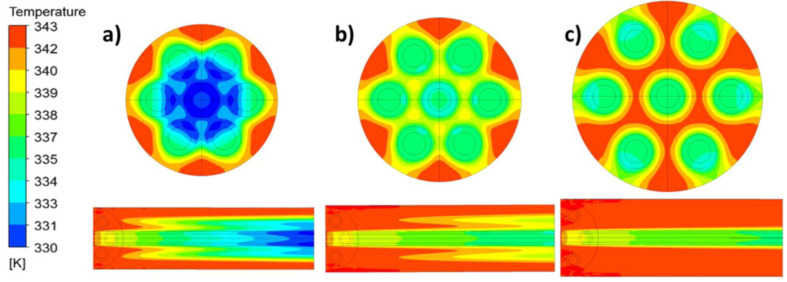
Temperature contours at the module outlet (cross-section) and from the inlet to outlet (axial profile) for module packing densities of (**a**) 2.4, (**b**) 1.8 and (**c**) 1.2 m^2^/m^3^ [53]. Reproduced with permission from Elsevier, 2016.

A numerical model for heat and mass transfer of VMD under laminar flow conditions using a CFD method was established [53]. Tests show that at high operating temperature (70 °C), a 56% increase in fiber packing density (Figure 18) leads to a 24% decrease in flow rate; at a low flow velocity (0.0072 m/s), the flow rate decreased by more than 50%. The decrease of permeate flux caused by fouling can be solved by a pretreatment process.

**Figure 19:**
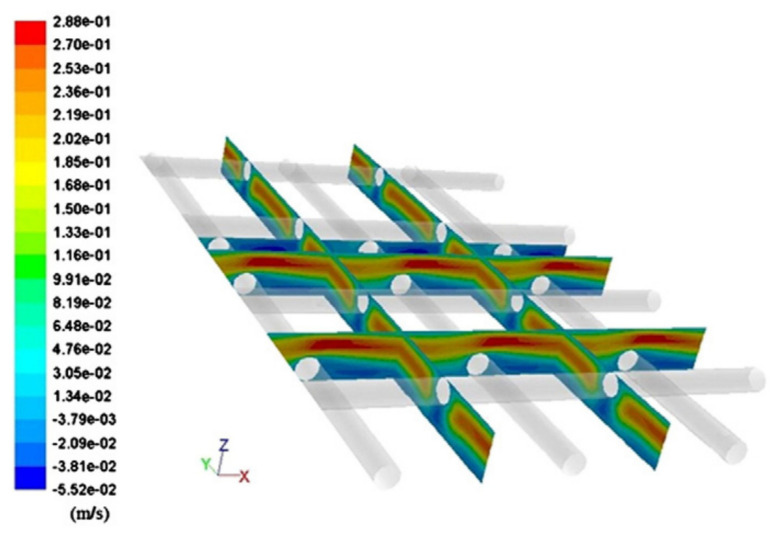
X-Velocity contours of the fluid flowing through the membrane [54]. Reproduced with permission from Elsevier, 2012.

Due to the existence of the feed spacers in the membrane module, it led to a secondary flow which enhanced the backwash, thus achieving the purpose of extending the service time of the membrane and increasing the efficiency of the membrane [54]. One example is shown in Figure 19.

A global sensitivity analysis technique was adapted to measure the effects of different parameters on permeate flux and the temperature polarization coefficient [55]. The results showed that the membrane thickness and feed temperature had the greatest effects on the design parameters in DCMD processes.

**Figure 20:**
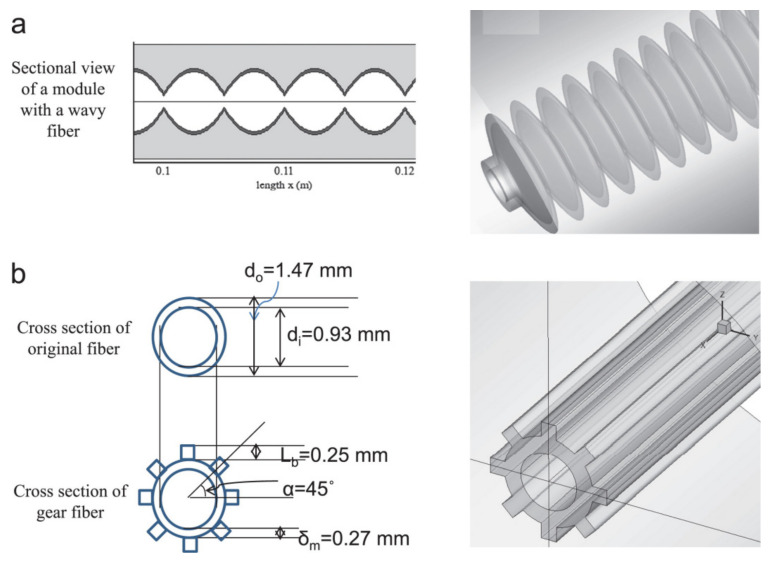
3D geometric structures of modules with (**a**) wavy fiber and (**b**) gear-shaped fiber [56]. Reproduced with permission from Elsevier, 2012.

In the literature [56], ten hollow-fiber membranes with different geometric structures were applied to a DCMD system and be evaluated the energy efficiency. The study shows that, under the equivalent effective length, the gear type module (Figure 20) has the highest flux enhancement rate, followed by the alternate-wave type module.

**Figure 21:**
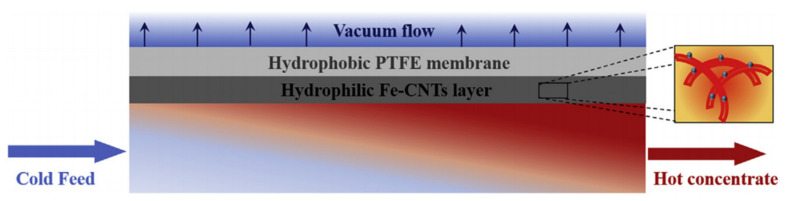
Illustration of the induction heated (IH)-VMD process. Bottom layer: temperature profile in the feed; Middle layer: composite membrane; Top: vacuum chamber. Right image: enlargement of the Fe-CNTs layer [50]. Reproduced with permission from Elsevier, 2019.

A new method for VMD using a high frequency magnetic field self-heating membrane was proposed [50], as shown in Figure 21. This self-heating system can overcome the temperature polarization and heat loss caused by a low permeate flux in VMD processes, and at the same time, it can desalinate a series of brine solutions. The system uses induction heating to heat the membrane surface quickly and directly, without contact, and without preheating a large amount of feed solution. With the increase of feed temperature at the feed side, the permeate flux at the permeation side increases.

In a previous study [57], the membrane module was simplified into a two-dimensional (2D) structure with a single membrane filament, thereby a 2D VMD model of NaCl aqueous solution under steady state was established using CFD. Then a series of studies were carried out on the CFD simulation of VMD, such as the designs of membrane modules, and the preparation of PP/polyolefin elastomer blend membranes, etc. The results show that volume fraction of vapor increased with increasing feed temperature. The vapor volume fraction reached a maximum of between 45 °C and 65 °C. Once the temperature reached 70 °C, the vapor volume fraction decreased. Under the same feed temperature, permeate flux of the VMD process increased with the increasing flow. 

**Figure 22:**
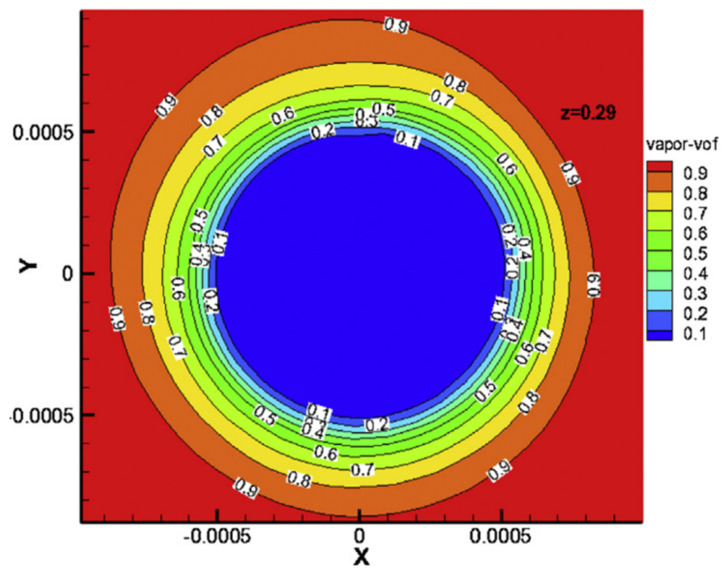
The vapor volume fraction changes at the position Z = 290 mm at a feed temperature of 333.15 K; velocity: 0.2 m/s [58]. Reproduced with permission from Elsevier, 2015.

Understanding the gas–liquid distribution in the membrane module is helpful when designing the membrane module and to enhance heat and mass transfer. As shown in Figure 22, the change of pressure and the change of liquid volume fraction mainly occur in the membrane filaments in the VMD process. The water vapor and liquid are distributed in the membrane filaments and the shell, respectively, and most of the changes of pressure, temperature and phase transition occur in the boundary layer of the membrane filaments [58].

## 5. Summary and Prospect

As shown in Table 1, CFD simulation has been widely used in MD process analysis, including; MD mechanisms and characteristics analysis, membrane module development, novel membrane preparation etc. A series of related research results have been achieved providing the solutions of temperature/concentration polarization and permeate flux enhancement. The physical phenomena and geometric structures have been greatly simplified in most CFD simulations of MD processes, so there remains much work to do in this field in the future. In addition, a great deal of attention has been paid to the hydrodynamics and heat transfer in the channels of MD modules, as well as the optimization of these modules. However, the study of momentum transfer, heat and mass transfer mechanisms in membrane pores is rarely involved. These projects should be combined with mass transfer, heat transfer and momentum transfer for a more comprehensive and in-depth research. In most CFD simulations of MD processes, some physical phenomena, such as surface diffusion, which occur in the membrane surface and have important guiding significance for the preparation of novel membranes should be studied further, but are mostly ignored. There are also some limitations of CFD in the simulation of the membrane process, including simulation of the membrane fouling process; high time consumption and the computer performance required in 3D simulations of MD. Moreover, some small-scale details in the membrane module may be ignored for simplifying simulation parameter settings.

As a result, although CFD simulation has been widely used in MD process modeling already, there are still problems that remain which require future study. It can be predicted that more complex mechanisms, such as permeable wall conditions, fouling dynamics, and multiple ionic component diffusion, will be included in the CFD modeling of MD processes. Furthermore, the users developed routines for MD processes will be incorporated into the existing commercial or open source CFD software packages.

## Figures and Tables

**Table 1:** Research topics and conclusions.

Research Contents		Conclusions
Mechanisms of different MD structures	DCMD	(1) The temperature polarization increases with the increase of the feed temperature and the decrease of the feed velocity and penetration velocity; The counter-current operation is better than the co-current operation in most cases, except for relatively low NaCl concentrations or sufficiently long module lengths [15]. (2) The DCMD process has a strong convective heat transfer mechanism, the main reason is that the temperature polarization and the Biot number in the DCMD processes are high. The membrane conduction resistance is dominant at very low flow rates, which limits the heat transfer at the membrane interface, thus affecting the process performance [8,14].
	VMD	(1) The feed temperature and vacuum degree highly affect operating variables for higher permeate flux and minimum specific energy consumption. The considerable reduction in the TPC value ranging from 0.81 to 0.48 was discovered in raising feed temperature from 25 °C to 85 °C, indicating a significant reduction in heat transfer resistance, resulting in a remarkable increase in permeate flux [16]. (2) The small-scale eddies induced by the presence of spacer filaments promote mixing in the module, thus the temperature and concentration polarization are alleviated, and the water vapor flux is immensely improved. The pressure drop by the spacers reduces the allowable module length by one order of magnitude, but the module length increases two folds at feed temperature of 80 °C [17]. (3) The volume fraction of vapor increased with increasing feed temperature; vapor volume fraction reached a maximum between 45 °C and 65 °C. Once the temperature reached 70 °C, the vapor volume fraction decreased. Under the same feed temperature, permeate flux of the VMD process increased with the increasing flow [57].
	AGMD	(1) The permeate flux can be increased by 4.70 and 5.46 times as much as that of DCMD after nitrogen and low-pressure water vapor are introduced. However, under the same experimental conditions, the permeation fluxes of water vapor with the same flow rate are all greater than that of nitrogen. The gas–liquid two-phase flow pattern with the same flow rate of nitrogen and low-pressure water vapor is obviously different from that of the feed liquid [18,19,20,21,22]. (2) In the momentum transfer process of AGMD, the turbulence intensity obviously increases. In the process of heat transfer of AGMD, the effect of the total temperature difference driving force is improved and the concentration polarization phenomenon is obviously weakened (the maximum temperature polarization factor of nitrogen and low-pressure water vapor can reach 1.64 times and 2.37 times of DCMD, while the concentration polarization factor can be reduced to 0.62 times and 0.22 times as low as that, without filling any bubble gas [18,19,20,21,22].
	SGMD	(1) The membrane thickness has a great influence on the SGMD performance, while the membrane porosity has a little influence on the SGMD performance [23,24].
Optimization analysis of membrane modules design		(1) Good feed spacer spacing helps to improve permeability. The small-scale eddy currents caused by the presence of spacers can promote liquid mixing in MD membrane modules, providing effective boundary layer surface renewal and a more uniform flow distribution, thereby reducing temperature and concentration polarization and increasing permeate flux [13,30,33,38]. (2) Adding spacer filaments in the membrane module will lead to increased power consumption [17,30]. (3) The thermal efficiency varies with the feed temperatures and feed velocities, so the temperature polarization phenomenon is more obvious at high feed temperatures and low feed velocities [31]. (4) The spacers in the membrane module increase the pressure drop and increase the heat transfer coefficient [32]. (5) The higher operating temperature can improve the heat and mass transfer and MD thermal efficiency even under relatively low temperature differences across the membrane [34]. (6) The orientation of the baffles has an effect on the temperature polarization and heat transfer rate. If the filaments contact the upper or lower surface of the membrane, the temperature polarization is very high, which is resulting in a lower heat transfer rate. When these filaments are separated from the membrane, the temperature polarization decreases [35]. (7) The permeate flux and thermal efficiency can be improved by increasing the flow rate and inlet temperature, and decreases the length of the membrane modules. Moreover, the temperature polarization coefficient distribution of the staggered filaments is more uniform, which indicates that this orientation is more suitable for enhancing the heat transfer in the MD process [37]. (8) The applications of the distributors significantly increase the permeation fluxes of the membrane modules. [42,43,44]. (9) Under laminar flows, the water production can be significantly improved by attaching baffles in the channel shells, which does not work under turbulent flows due to the huge increase in hydrodynamic loss [38]. (10) The mass transfer coefficient stays nearly unchanged for different baffled modules. The impacts of baffles on the water production lies in the transmembrane partial vapor pressure difference. Baffles impact the CPC and TPC, and thus the transmembrane partial vapor pressure difference [38].
Preparation of novel membranes and analysis of membrane characteristics		(1) Self-heating membranes improve the transmembrane driving force. [4,50]. (2) The optimal membrane thickness is between 10–20 µm, which corresponds to the selected materials, pore size distribution and the maximum permeability flux under the operating conditions. The membrane materials with low thermal conductivity and high porosity are easily obtain higher permeation flux and larger optimal membrane thickness, while the optimal membrane thickness can be reduced by increasing the feeding speed or temperature [15]. (3) The probability of membrane fouling can be determined though the velocity distribution [52]. (4) At high operating temperatures (70 °C), a 56% increase in fiber packing density leads to a 24% decrease in flow rate; At low flow velocity (0.0072 m/s), the flow rate decreased by more than 50%. The decrease in permeate flux caused by fouling can be solved by a pretreatment process [53]. (5) The existence of the feed spacers in the membrane module will lead to a secondary flow which will enhance backwash, thus achieving the purpose of extending the service time of the membrane and increasing the efficiency of the membrane [54]. (6) The membrane thickness and feed temperature had the greatest effects on the design parameters [55]. (7) Under the equivalent effective length, the gear type module has the highest flux enhancement rate, followed by the alternate-wave type module [56]. (8) The change of pressure and the change of liquid volume fraction mainly occur in the membrane filaments, the water vapor and liquid are distributed in the membrane filaments and the shell respectively, and most of the changes of pressure, temperature and phase transition occur in the boundary layer of the membrane filaments [58].
